# Multiphysics and Thermal Response Models to Improve Accuracy of Local Temperature Estimation in Rat Cortex under Microwave Exposure

**DOI:** 10.3390/ijerph14040358

**Published:** 2017-03-30

**Authors:** Sachiko Kodera, Jose Gomez-Tames, Akimasa Hirata, Hiroshi Masuda, Takuji Arima, Soichi Watanabe

**Affiliations:** 1 Department of Electrical and Mechanical Engineering, Nagoya Institute of Technology, Nagoya 466-8555, Japan; jgomez@nitech.ac.jp (J.G.-T.); ahirata@nitech.ac.jp (A.H.); 2 Department of Environmental Medicine, Kurume University School of Medicine, Fukuoka 830-0011, Japan; bdxmsd@yahoo.co.jp; 3 Department of Electrical and Electronics Engineering, Tokyo University of Agriculture and Technology, Tokyo 184-8588, Japan; t-arima@cc.tuat.ac.jp; 4 Electromagnetic Compatibility Laboratory, Applied Electromagnetic Research Institute, National Institute of Information and Communications Technology, Tokyo 184-8795, Japan; wata@nict.go.jp

**Keywords:** temperature elevation, thermophysiology, finite-difference time-domain method, bioheat equation, electromagnetic field

## Abstract

The rapid development of wireless technology has led to widespread concerns regarding adverse human health effects caused by exposure to electromagnetic fields. Temperature elevation in biological bodies is an important factor that can adversely affect health. A thermophysiological model is desired to quantify microwave (MW) induced temperature elevations. In this study, parameters related to thermophysiological responses for MW exposures were estimated using an electromagnetic-thermodynamics simulation technique. To the authors’ knowledge, this is the first study in which parameters related to regional cerebral blood flow in a rat model were extracted at a high degree of accuracy through experimental measurements for localized MW exposure at frequencies exceeding 6 GHz. The findings indicate that the improved modeling parameters yield computed results that match well with the measured quantities during and after exposure in rats. It is expected that the computational model will be helpful in estimating the temperature elevation in the rat brain at multiple observation points (that are difficult to measure simultaneously) and in explaining the physiological changes in the local cortex region.

## 1. Introduction

In recent years, microwave (MW) has been used for various purposes such as diagnostic imaging, hyperthermia therapy, and telecommunications. The rapid development of wireless technology has changed electromagnetic environment around humans, and led to widespread concerns regarding adverse human health effects caused by exposure to electromagnetic fields (EMFs). International standardization bodies, such as the International Commission on Non-Ionizing Radiation Protection (ICNIRP) [1] and the Institute of Electrical and Electronics Engineers (IEEE) [2], have established guidelines for EMF exposures. The standards/guidelines indicate that the temperature elevation causes a dominant effect of EMF exposures at frequencies exceeding 100 kHz, although some studies have explored the possibility of nonthermal effects. Many studies examine thermal damages in animals and extrapolate the findings to humans, primarily for ethical reasons concerning health risks to human subjects [3,4,5], as also summarized in the guidelines/standards. Animal studies are one of the important topics in safety assessment as reviewed [6,7]. In most animal studies, specific absorption rates (SARs, power absorption per unit mass) are evaluated computationally to estimate the heat load. In experiments, the number of locations for measuring the temperature is limited. Thus, it is useful to develop a computational model for multiphysics (electromagnetics and thermodynamics) and thermal response to follow temperature and blood change for microwave (MW) exposure, particularly for intense localized MW exposure. To date, only a few computational models considering multiphysics and thermal response have been developed (e.g., rats [5,8,9] and rabbits [4]).

In our previous study, we designed an exposure apparatus for rat brain exposure [10], and then measured the thermophysiological response [11,12]. The thermoregulatory response was characterized in terms of temperature change with respect to local brain and body core temperatures [4,13]. We also developed a code by combining the multiphysics model with the thermophysiological response considered at a frequency of 1.5 GHz [9] associated with mobile phone signals. The study involved proposing a thermoregulatory response model in the rat brain based on local brain and body core temperatures. However, rat dimensions are notably less than those of the antennas in the frequency band, and thus the temperature increased in the regional brain tissue, which was exposed locally, as well as throughout the body (including the body core).

An apparatus for highly-localized MW exposure employing frequencies higher than several GHz is developed in the present study to maximize tissue exposure local to the antenna, while suppressing exposure levels elsewhere in the rat body. This improves the accuracy of the thermophysiological model and makes it suitable for general applications. Specifically, it is useful for a number of applications to evaluate thermal evaluation in a consistent model that can be used for conventional and higher frequencies. Such a model can be a powerful tool in providing useful data when replicating previous measurements, some of which provide a rationale of MW protection guidelines/standards. For intense localized exposure, thermal damages, including facial burning and cataract, are investigated in different animals [14,15]. Note that owing to the similarity of thermophysiology with humans, the data of primates are particularly important for whole-body exposure [16].

The aim of this study is to simulate the temperature elevation in rat brains experiencing localized exposure to high-intensity MW by considering heat transfer and thermophysiology. First, parameters that accurately reflect changes in regional cerebral blood flow (rCBF) were extracted from experimental data using live rats. Next, these parameters were incorporated into our thermophysiological model to simulate the temperature elevation during and after local MW exposure. Finally, the effectiveness of the newly acquired computational model was examined by comparing the temperature changes simulated by the model with the measured values.

## 2. Materials and Methods

### 2.1. Animal Experiments

Changes in physiological parameters were measured using live rats under MW exposure corresponding to three frequencies (1.5, 6, and 10 GHz). All animal experiments corresponding to 6 and 10 GHz were approved by the Institutional Animal Care and Use Committee of Tokyo Medical and Dental University (No. 0140287A, 0150285A, 0160322A). The animal experimental data corresponding to 1.5 GHz were obtained in a previous study [9] and reused to evaluate the effectiveness of the computational model proposed in the present study.

#### 2.1.1. Animals

Eight-week old male Sprague-Dawley rats (Japan SLC, Inc., Shizuoka, Japan) were used in the experiments. The rats were fed a standard pellet diet and given water ad libitum in an animal room with a 12 h light/dark cycle at a temperature corresponding to 23.0 ± 1 °C and relative humidity corresponding to 50% ± 20%.

#### 2.1.2. Definition of the Target Area

The target area is defined as a disk-shaped area of a rat’s right parietal cortex tissue (4 mm in diameter and 0.25 mm deep), within which physiological responses to MW exposure were evaluated. The target area is located immediately under the dura mater, and its center is located 4 mm posterior to the bregma and 4 mm to the right of the midline.

#### 2.1.3. Measurement of Physiological Parameters

Two physiological parameters, temperature of the rectum and target area and rCBF in the target area, were simultaneously measured using methods described in our previous study [9,17]. Briefly, the rats were anesthetized during the experiment and were placed on a heated pad after the head was fixed in the acrylic stereotaxic apparatus. The temperature of the heated pad was kept at 42 °C for a 1.5 GHz-exposure system, and at 39 °C for 6 and 10 GHz-exposure systems to stabilize temperatures in each measured region under sham-exposure conditions in our experimental setup. The right parietal skin was locally excised, and the skull of the right hemisphere was exposed. Three independent holes (0.5 mm in diameter) were drilled into the skull just above the target area 1.0 mm apart from each other. Two optical fibers (0.2 mm in diameter) connected to the Doppler blood flow meter (FLO-C1, Omegawave, Tokyo, Japan) and one optical probe (0.5 mm in diameter) connected to a thermometer (m600, Luxtron, Santa Clara, CA, USA) were independently inserted through the three holes and placed on the dura mater above the target area. Another thermometer probe was inserted into the rectum. The above-mentioned probes are all made of nonmetallic material. The blood flow and temperature signals were recorded at a 1.0 Hz sampling rate through an A/D converter and time was averaged over 1 min intervals.

#### 2.1.4. The 1.5 GHz Experimental System

The animal experimental corresponding to 1.5 GHz performed and described in the previous study [9]. As reference information, the rat brain was locally exposed to 1457 MHz MW by using a figure-8 loop antenna. The rats were anesthetized using an intramuscular injection of ketamine (100 mg·kg^−1^) and xylazine (10 mg·kg^−1^) in conjunction with a subcutaneous injection of pentobarbital (12.5 mg·kg^−1^). The rat head was immobilized in an acrylic stereotaxic apparatus and placed in the manipulator system fitted with an antenna positioned 4 mm over the target area. The radiation power of the antenna corresponded to 3.4 W. The corresponding specific absorption rate averaged over the brain (BASAR) was 75 W·kg^−1^. BASAR was calculated using the numerical rat model (described as follows). The total duration of the MW exposure corresponded to 6 min and followed the premeasurement of physiological parameters. The parameters were measured in the exposed rats (*n* = 4) and the sham-exposed rats (*n* = 4), which were prepared according to the same system but without MW exposure (0 W·kg^−1^ of BASAR).

#### 2.1.5. The 6 GHz and 10 GHz Experimental Systems

A microstrip-fed monopole antenna was used for an ultra-wideband exposure of the rat brain at 6 and 10 GHz [18]. The rats were anesthetized with 3% isoflurane in O_2_ and maintained at 2% isoflurane in O_2_ throughout the experiment. The rat head was immobilized in an acrylic stereotaxic apparatus. The antenna was positioned 5 mm over the right parietal bone immediately above the target area. Two exposure intensities were used at each frequency. The radiation power of the antenna corresponded to 1.5 and 2.4 W at 6 GHz, and 0.8 and 2.4 W at 10 GHz. The computed BASARs in the 6 GHz experiment corresponded to 64 and 106 W·kg^−1^, and those in the 10 GHz experiment corresponded to 29 and 79 W·kg^−1^. The total duration of the MW exposure corresponded to 18 min and followed the premeasurement of physiological parameters conducted for 6 min. Four rats were prepared for each exposure condition, including the sham-exposure.

Note that the metric in the safety guidelines is SAR averaged over 10 g of tissue in the safety guidelines [4,19]. As mentioned in World [20], this physical quantity provides good correlation with the temperature elevation. However, this is a metric used for localized exposures for frequencies up to 3 GHz in IEEE-C95.1 [2] and 10 GHz in ICNIRP [1]; in addition, a 10-g volume is difficult for use in small animals. In other words, no metric exists for the thermal effect over wide frequencies. Thus, BASAR (mass of the brain of 1.9 g) was used as an empirical measurement because of the lack of metric.

### 2.2. Numerical Rat Model and Exposure Scenarios

**Figure 1:**
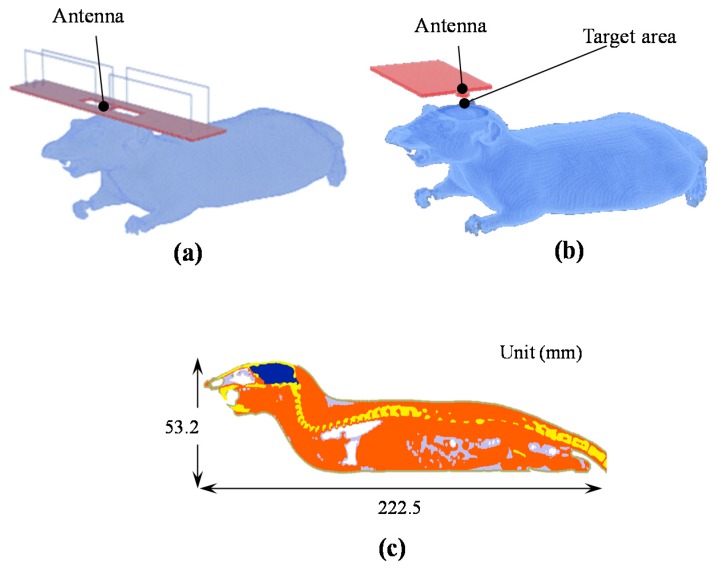
Position of the rat and antennas during exposure: (**a**) the antennas correspond to a figure-8 loop antenna for a 1.5 GHz exposure; (**b**) monopole-fed antenna for 6 and 10 GHz exposure; and (**c**) the cross section and size of the numerical rat model.

A numerical rat model was developed from CT images, as seen in Figure 1c [10]. The model is composed of the following six tissues: muscle, skin, fat, eye, bone, and brain. The voxelized model (94,276,000 elements) has a resolution of 0.25 mm. The skin that covered the parietal bones was removed to compare with the animal experiment in the same condition. The whole-body and brain weights used in the rat model corresponded to 265 and 1.9 g, respectively. The measuring probes for temperature and rCBF possess a sufficiently small diameter below 0.5 mm, and all the probes are composed of quartz optical fiber (ε*_r_* = 3.7, σ = 1 × 10^−18^ S·m^−1^). Thus, the probes did not significantly violate the electromagnetic field distribution. The probes were replaced with air in the simulation.

### 2.3. SAR Computation

The finite-difference time-domain (FDTD) method was used to calculate the MW power absorbed in the numerical rat model [21]. The SAR is defined as follows:

(1) $$SAR(r)=\frac{\sigma (r)}{2\rho (r)}{\left|E(r)\right|}^{2}$$

where ｜**E**(**r**)｜ denotes the peak value of the electric field at position **r**, and *σ*(**r**) and *ρ*(**r**) denote the conductivity and mass density of the tissue, respectively. Table 1 shows the dielectric properties used in the model, acquired from Reference [22]. The perfectly matched layers were used as the absorbing boundary condition for absorbing outgoing scattered waves to simulate an infinite space [23]. Time discretization was determined at 4.77 × 10^−13^ s, that is, 0.99 times the limit of the Courant condition [21]. The accuracy of the SAR assessment was confirmed by Arima et al. [10]. Note that the value of SAR is assumed to be time-invariant as it is proportional to the square of the electric field peak, and the rat was immobilized in the exposure system. The metric BASAR was computed as the SAR averaged over the brain in a numerical rat model for 1.5, 6, and 10 GHz for each radiation power of the antenna used in this study.

### 2.4. Temperature Computation

#### 2.4.1. Bioheat Equation

After the formulation of Pennes’ bioheat equation [24], many improvements have been made to it for different purposes. Bioheat equations are often used to calculate temperature elevation in numerical models. The equation provides approximate temperature and is demonstrated to be a reasonable and powerful tool for analyzing temperature, especially in the shallow region of the body [25,26]. However, it does not satisfy the first law of thermodynamics as the heat removed from tissue with blood perfusion vanishes in a conventional formulation of the bioheat equation. This violation would not be essential when the radio frequency (RF) energy deposition in the body is smaller than the total amount of the basal metabolism. For intense localized exposure (as in this study), the body-core temperature elevates with time (e.g., see [27]; rabbits exposed to intense localized exposures). To analyze the temperature in the body, Bernardi et al. [28] treated the blood temperature in the bioheat equation as variable over time but constant over the body to consider the energy deposited in the blood. We improved the formula for blood temperature in the voxelized models [29]. The rationale for considering blood temperature as constant over the body is that the blood in large vessels circulates throughout the human body in a minute or less [30]. The blood circulation time in rats is much smaller than that in humans because of their smaller body and larger blood perfusion rate.

The bioheat equation considers heat exchange mechanisms, including heat conduction and blood perfusion, as follows [28]:

(2) $$C\left(r\right)\rho \left(r\right)\frac{\partial T\left(r,t\right)}{\partial t}=\nabla \cdot \left(K\left(r\right)\nabla T\left(r,t\right)\right)+\rho \left(r\right)SAR\left(r\right)+A\left(r,t\right)-B\left(r,t\right)\left(T\left(r,t\right)-T_{B}\left(r,t\right)\right)$$

where **r** and *t* denote the tissue position vectors and time, respectively; and *T*(**r**,*t*) and *T_B_*(**r**,*t*) denote the temperatures of tissue and blood, respectively. Additionally, *C*(**r**) denotes specific heat of tissue, *K*(**r**) denotes thermal conductivity of the tissue, *A*(**r**,*t*) denotes metabolic heat, and *B*(**r**,*t*) denotes the factor related to the blood perfusion. SAR determined from Equation (1) is substituted in the bioheat equation as a heat source.

The radiation boundary condition between tissues and external air is as follows:

(3) $$-K\left(r\right)\frac{\partial T\left(r,t\right)}{\partial n}=H\left(r\right)\cdot \left(T\left(r,t\right)-T_{a}(t)\right)$$

where *T_a_*(*t*), *H*(**r**), and **n** denote ambient temperature, heat transfer coefficient, and a vector normal to the body surface, respectively. The ambient temperature in the lung is approximated using the average temperatures of blood and ambient air temperature. The ambient air temperature was controlled in the experiment to 23 °C. Note that the heat diffusion length in the biological tissue is on the order of 7 mm (skin) [31], and thus this modeling does not affect local temperature change in the head. Although this modeling may affect the body core temperature, we assumed heating by using a warm pad to compensate for the reduction in basal metabolic rate due to the systemic anesthesia. In our study, the heat transfer coefficient is also chosen to satisfy the thermal equilibrium, and thus the discretization error discussed in Reference [32] is implicitly considered. This may also not affect the core temperature as well.

The volume-averaged blood temperature is changed so as to satisfy the first law of thermodynamics. The blood temperature is changed as follows [28,33]:

(4) $$T_{B}\left(t\right)=T_{B0}+\int_{t}\frac{Q_{BT}\left(t\right)-Q_{BT}\left(0\right)}{C_{B}\rho_{B}V_{B}}dt$$

(5) $$Q_{BT}\left(t\right)=\int_{V}B\left(t\right)\left(T_{B}\left(t\right)-T\left(r,t\right)\right)dV$$

where *Q_BT_*(*t*) denotes the total receiving heat quantity from tissues to blood. Additionally, *C_B_* (=4000 J·kg^−1^·°C^−1^), *ρ_B_*(=1058 kg·m^−3^), *T_B_*_0_, and *V_B_* denote the specific heat of blood, mass density of blood, initial blood temperature, and total volume of blood, respectively. The average blood volume per unit of rat body mass correspond to 64 mL·kg^−1^ [34]. The blood volume of the rat model was set at 18.9 mL.

The initial temperature *T*_0_ was solved using Equations (2) and (3) by setting *SAR* = 0 W·kg^−1^ and *T_B_*_0_ = 37.0 °C. *T_B_*_0_ was estimated from the average rectal temperature (body core temperature) of the anesthetized live rats without exposure. The bioheat equation subject to the boundary condition was solved to obtain the thermally steady-state temperature elevation. Thus, the left-hand-side term of Equation (2) was assumed as zero.

#### 2.4.2. Blood Flow

The blood flow parameter *B*(**r**,*t*) in the skin depends on both the hypothalamus temperature and local skin temperature [35]. Skin blood flow was regulated by hypothalamus temperature, and the average temperature of the skin can be expressed as follows:

(6) $$\begin{array}{l} B\left(r,t\right)=\left[B_{0}\left(r\right)+F_{HB}\left(T_{H}\left(t\right)-T_{H0}\right)+F_{SB}\bar{\Delta T_{S}}\right]\cdot 2^{\left(T\left(r,t\right)-T_{0}\left(r\right)\right)/6}\\ \bar{\Delta T_{S}}=\int_{S}\left(T\left(r,t\right)-T_{0}\left(r\right)\right)dS/S, \end{array}$$

where *B*_0_(**r**) denotes basal blood flow of each tissue, $\bar{\Delta T_{S}}$ denotes average temperature elevations of skin, *S* denotes the skin area, and (*T_H_*(*t*) − *T_H_*_0_) denotes an elevation of hypothalamus temperature. Additionally, *F_HB_* (=17,500 W·m^−3^·°C^−1^) and *F_SB_* (=1100·W·m^−3^·°C^−1^) correspond to coefficients that determine the changes in the blood flow characteristics over time [33]. In this study, the rat brain had a strong heat source and the hypothalamus was not modeled in detail. Thus, the blood temperature elevation was used as a body core temperature as opposed to the hypothalamus values.

The regulation of cerebral blood flow is expressed in Equation (2) [9]. Blood flow regulation in the brain was based on the measured blood flow rates in the experiment. The cerebral blood flow parameter is considered to be influenced by the rectum and brain temperature elevations (based on the hypothesis that thermal regulation in living organisms contributes to temperature changes in body core and local parts); this is expressed as follows:

(7) $$B\left(r,t\right)=B_{0}\left(r\right)\cdot \left(1+F_{RB}\cdot \left(T_{R}\left(t\right)-T_{R0}\right)\right)\cdot 2^{\left(T\left(r,t\right)-T_{0}\right)/F_{BB}}$$

where *T_R_*(*t*) denotes rectal temperature and *F_RB_* and *F_BB_* denote weighting coefficients in relation to the variation of rectal and brain temperature elevations, respectively. The rectal tissue was not modeled in detail, and thus *T_R_*(*t*) is replaced by the blood temperature in the simulation. The rectum has a large blood volume, and thus it is a suitable approximation for this work [36].

Blood flow in tissues except of the skin and brain is regulated as follows [37,38]:

(8) $$\begin{array}{l} B\left(r,t\right)=B_{0}(r),\\ B\left(r,t\right)=B_{0}(r)\left[1+S_{B}\left(T\left(r,t\right)-39\right)\right],\\ B\left(r,t\right)=B_{0}(r)\left[1+5\cdot S_{B}\right], \end{array}\begin{array}{l} T\left(r,t\right)\leq 39{}^{\circ }C\\ 39{}^{\circ }C\leq T\left(r,t\right)\leq 44{}^{\circ }C\\ 44{}^{\circ }C\leq T\left(r,t\right), \end{array}$$

where *B*_0_(**r**) is based on the blood flow in each tissue and *S_B_* (=0.8 °C^−1^) denotes the coefficient for determining the changes in the blood flow characteristics over time.

#### 2.4.3. Thermal Constants

Table 2 lists the thermal parameters of tissues adapted in the rat model. The specific heat and thermal conductivities shown in a study by Cooper et al. [39] were used. The term associated with blood flow for each tissue was extrapolated from a study by Gordon et al. [40]. The basal metabolism of the entire body of a rat was estimated based on Kleiber’s law [41] and associated with each tissue in the model based on a reference to the proportion of blood flow [40]. The blood flow for eye tissue was ignored for simplicity. Not that blood flow on the retina and choroid is high. However, it has minor impact on the temperature elevation in the target area.

The heat transfer coefficients were based on those used in a study by Hirata et al. [9]; the coefficient of air and skin is 0.5 W·m^−2^·°C^−1^ and that of lung and air is 8.1 W·m^−2^·°C^−1^. For these parameter sets, together with the basal metabolism in Table 2, the heat balance between the air and rat model is maintained (basal metabolism of the rat model was estimated at 1.4 W).

#### 2.4.4. Evaluation Index for Effects of Thermoregulation

To confirm the effects of thermoregulatory modeling on the temperature in the brain, the temperature is computed under the assumption that the blood flow in the tissues is constant at *B*_0_, as shown in Table 2. Note that this case is termed as “without thermoregulation” in Table 3. The evaluation index I is defined as follows:

(9) $$\begin{array}{l} I=\bar{\Delta T_{m,c}}/T_{\max\_TA\_m}\\ \bar{\Delta T_{m,c}}=\int \left|T_{TA\_c}(t)-T_{TA\_m}(t)\right|dt/t_{duration}, \end{array}$$

where *T_TA_c_*(*t*) and *T_TA_m_*(*t*) denote the computed and measured temperature elevations in the target area, *t_duration_* denotes the total duration of measurement, and *I* denotes the time average of the difference between the measured and computed temperature elevations ($\bar{\Delta T_{m,c}}$) normalized by the maximum value of the measured temperature elevation (*T_max_TA_m_*) during the measurement.

#### 2.4.5. Data Analysis

Statistical analysis was done using the Mann–Whitney U test to evaluate the difference in regional temperatures between before and after MW exposure. A *p* value of <0.05 was considered statistically significant.

## 3. Results

### 3.1. Time-Course of Changes in Physiological Parameters

**Figure 2:**
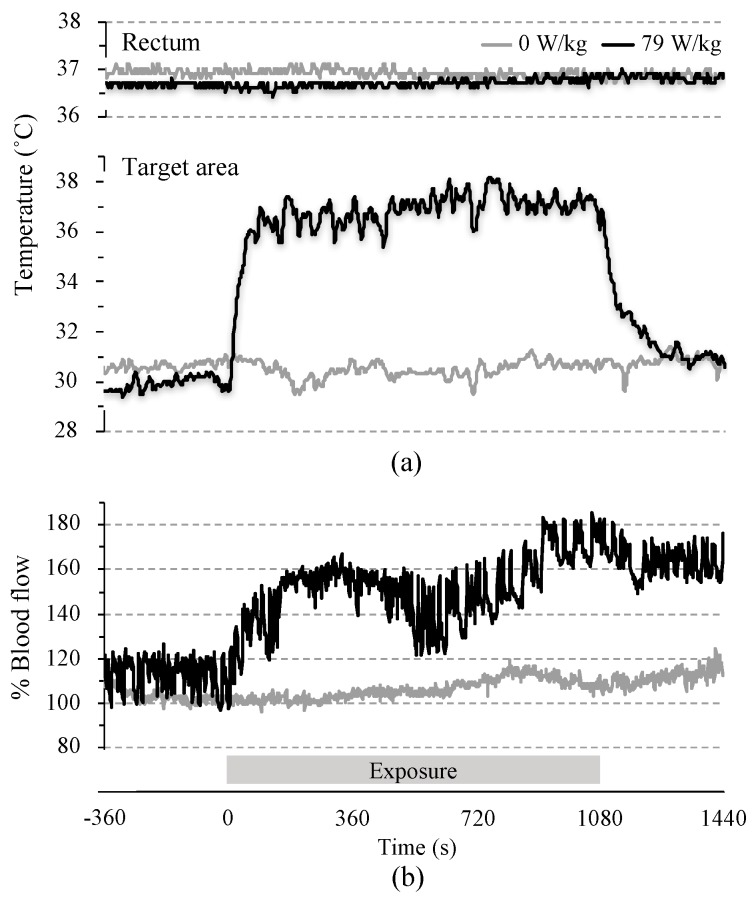
Typical responses to localized microwave exposure. Three physiological parameters: (**a**) rectal and target area temperatures; and (**b**) regional cerebral blood flow in target area, were simultaneously measured before, during, and after the localized 10 GHz MW exposure. The physiological parameters were obtained under sham-exposed and exposed rats (*n* = 1 each).

To investigate whether regional temperatures and rCBF were modified by MW exposure, we measured these parameters simultaneously during the exposure. Figure 2 shows a typical response to 10 GHz-MW exposure at 0 and 79 W/kg BASAR. Parameters, such as rectal temperature, target area temperature, or rCBF, showed unremarkable changes in sham-exposed conditions. In contrast, the target area temperature rose immediately after the beginning of the exposure, whereas the rectal temperatures increased slightly (<0.3 °C) until the end of the exposure. An increase in rCBF also appeared immediately after the beginning of the exposure period and lasted until the end of the experiment.

### 3.2. Temperature Conditions in Animal Experiments

Table 4 shows practical temperatures in two regions at the beginning and end of the MW exposure in the animal experiments. Before the exposure, the basal temperatures in the rectum and target area were 36.4–37.5 °C and 29.4–33.3 °C, respectively. To examine the stability of heat balance in our experimental setup and anesthetized animals, the temperatures in two regions, that is, before and after the sham exposure were compared. The result showed no significant difference in either temperature for any experimental setup. In contrast, a significant temperature elevation was found in the target area after the MW exposure. However, even maximum temperature elevation was less than 38.0 °C because the initial temperature in the target area was around 31 °C.

### 3.3. Extraction of Parameters Associated with the Blood Flow Rate in the Brain

The determination of *F_BB_* and *F_RB_* is required to compute the temperature elevations in the target area of the rat brain, considering the thermoregulation. First, the experimental results for rats under exposure corresponding to 10 GHz including the BASARs of 29 and 79 W·kg^−1^ (*n* = 7) were selected, in which rectal temperature did not elevate, and thus the effect of the rectal temperature elevation was neglected. Additionally, *F_BB_* was estimated using least-squares regression between the time course of rCBF and the brain temperature. The *F_BB_* parameter corresponds to 14 °C.

The value of *F_BB_* was used to estimate *F_RB_* by employing least-squares regression between the time course of rCBF and the temperature at the rectum and target area under MW exposure of 6 GHz including BASARs corresponding to 38, 64, and 106 W·kg^−1^ (*n* = 8). *F_RB_* corresponded to 0.053 °C^−1^.

The values of *F_BB_* and *F_RB_* determined in a previous study [9] were 31 °C and 0.050 °C^−1^ from measurements performed at 1.5 GHz MW exposure. For the purposes of comparing the parameters, the temperature elevation in the target area was computed and compared using index *I* (Equation (9)). The values of *I* for the 106 W·kg^−1^ BASAR at 6 GHz and 79 W·kg^−1^ BASAR at 10 GHz corresponded to 9.2% and 9.1%, which exceeded 4.7% and 5.3%, respectively, as obtained using the parameter values derived in this study. Thus, *F_BB_* and *F_RB_* correspond to 14 °C and 0.053 °C^−1^, respectively, as discussed subsequently.

**Figure 3:**
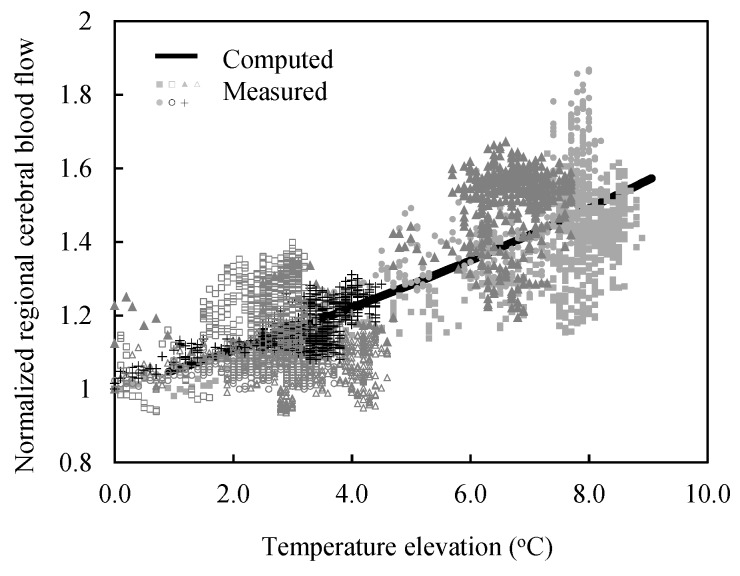
Regional cerebral blood flow and regional temperatures in the target area of the rat brain during localized MW exposure at 10 GHz. The solid line shows the values calculated based on Equation (7). The markers show the experimental values (*n* = 7, depicted by the markers) including the SARs averaged over the brain that correspond to 29 and 79 W·kg^−1^.

Figure 3 shows the correlation between the changes in rCBF and temperature elevation in the target area exposed to 10 GHz at 29 and 79 W·kg^−1^ of BASARs. The measured rCBF exhibited temporal fluctuations with the heartbeat and vasomotor and a positive correlation with the temperature.

### 3.4. Effects of Thermoregulation

**Figure 4:**
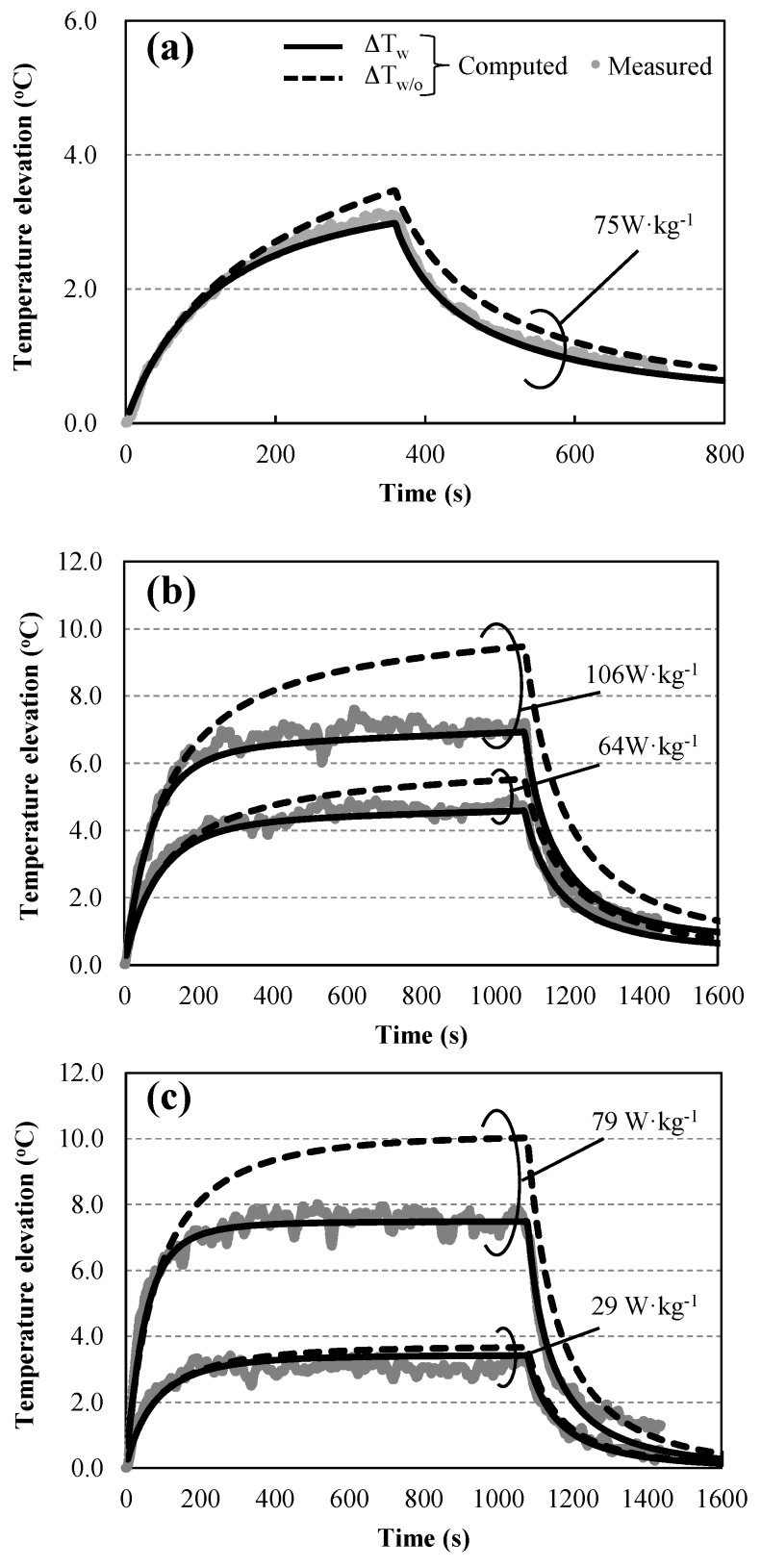
Comparison of temperature elevations with and without thermoregulation at different specific absorption rates averaged over the brain (BASARs): (**a**) 1.5 GHz; (**b**) 6 GHz; and (**c**) 10 GHz. The solid line shows the computed temperature with thermoregulation at the central target area (Δ*T_W_*). The broken line shows the computed temperature without thermoregulation (Δ*T_W/O_*). Average values of measured temperatures are plotted for comparison with computed values.

Figure 4 shows the estimation of time course in temperature changes at target area with and without consideration of thermoregulation parameter for different exposure intensities and frequencies. As shown in Figure 4, the difference in temperature elevation between the target areas with and without thermoregulation modeling increased with corresponding increase in the value of BASAR. Next, the index *I* (Equation (9)) was calculated using the data in Figure 4. Table 3 shows the comparison between the temperature elevations in the target areas with and without thermoregulation modeling that correspond to *I_ΔT_W_* and *I_ΔT_W/O_*, respectively. The index *I_ΔT_W_* was lower than *I_ΔT_W/O_* across all the conditions.

### 3.5. Computation of the SAR and Temperature Distributions in the Numerical Rat Model

**Figure 5:**
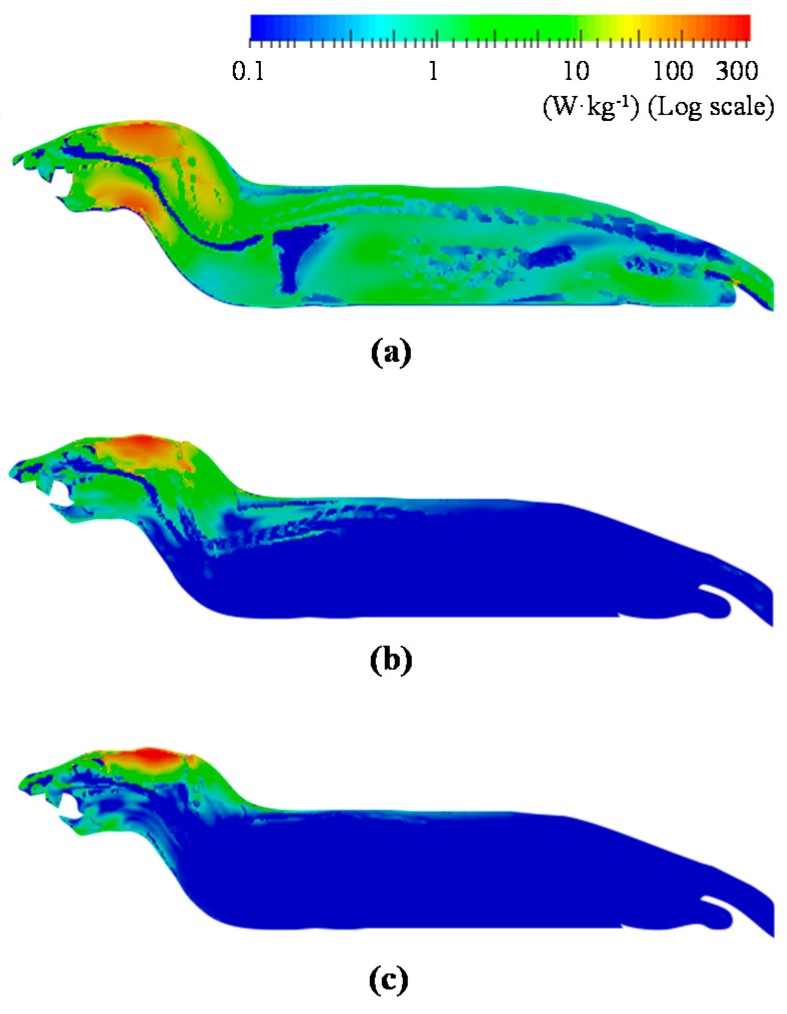
Distributions of SAR: (**a**) 1.5 GHz; (**b**) 6 GHz; and (**c**) 10 GHz. The BASARs correspond to: (**a**) 75 W·kg^−1^; (**b**) 64 W·kg^−1^; and (**c**) 79 W·kg^−1^. Exposure durations correspond to: 6 min in (**a**); and 18 min (**b**,**c**).

Figure 5a–c shows the SAR (Equation (1)) distribution under MW exposure at 1.5, 6, and 10 GHz, respectively. The maximum SAR computed in all models was located approximately near the parietal region; this corresponded to the position immediately below the antennas. Specifically, the absorption region of SAR was localized at the surface. The BASARs at 1.5, 6, and 10 GHz were 16, 32, and 30 times larger than the whole-body average SARs (WBASAR), respectively. These results suggested that MW power was mainly absorbed approximately near the parietal region with the exception of 1.5 GHz because the SAR was high even at the throat.

**Figure 6:**
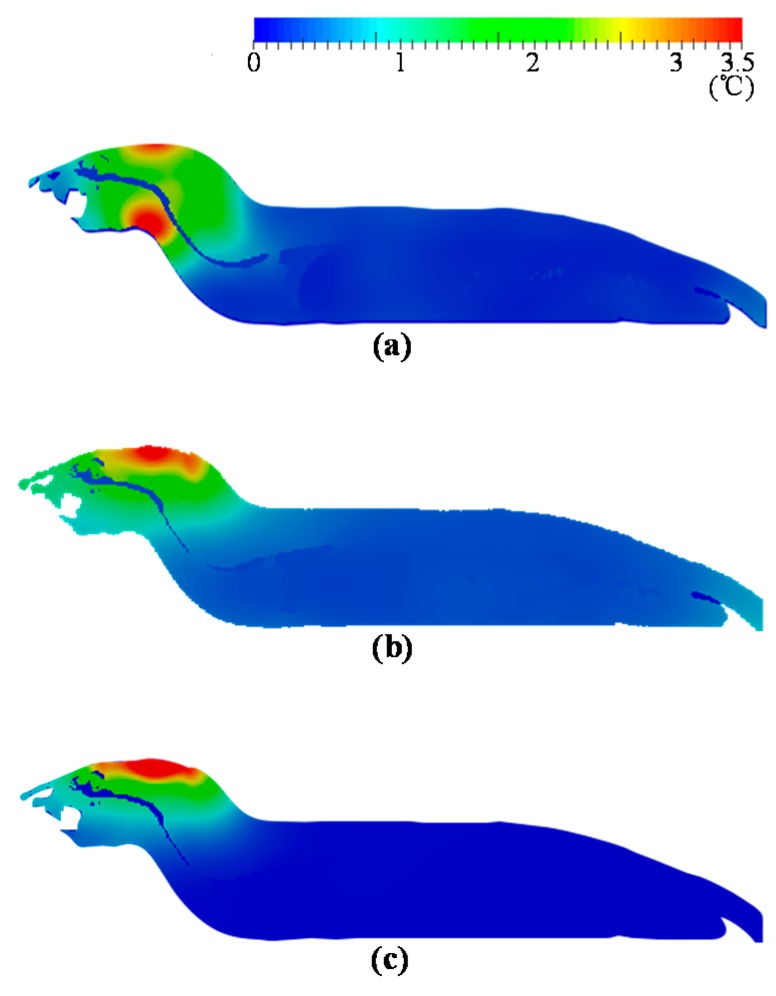
Distributions of maximum temperature elevation: (**a**) 1.5 GHz; (**b**) 6 GHz; and (**c**) 10 GHz. The BASARs correspond to: (**a**) 75 W·kg^−1^; (**b**) 64 W·kg^−1^; and (**c**) 79 W·kg^−1^. Exposure durations correspond to: 6 min in (**a**) and 18 min (**b**,**c**).

Figure 6a–c shows the distribution of temperature elevations at 1.5, 6, and 10 GHz, respectively. The distribution following 6 min of exposure is shown at 1.5 GHz, whereas the distributions following 18 min of exposure are shown at 6 and 10 GHz. The distribution patterns of temperature elevation were similar to those of the SAR albeit with less spatial variation. The maximum temperature elevation appeared in the target area of the brain at 6 and 10 GHz, whereas the maximum temperature elevation appeared around the throat at 1.5 GHz.

### 3.6. Comparison of Temperature Elevations Between Live Rats and Numerical Rat Models

**Figure 7:**
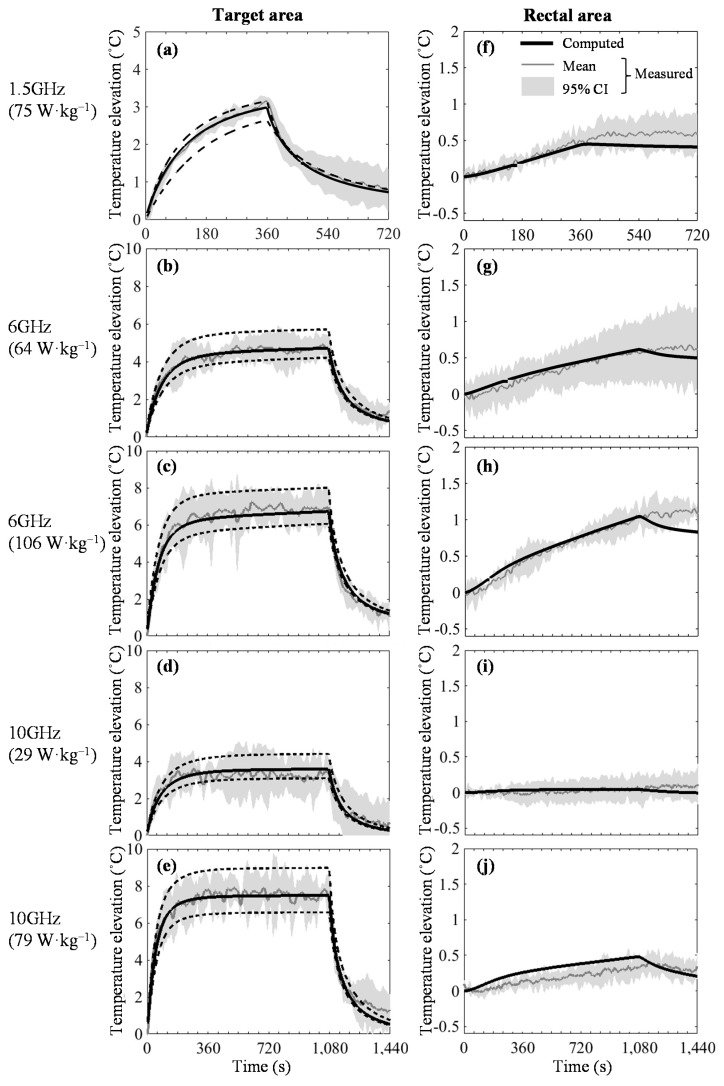
Temperature elevations in the target and rectal areas for each BASAR. Exposure durations correspond to: 6 min (**a**,**f**); and 18 min (**b**–**e**,**g**–**j**). The black bold line shows the temperature elevation computed in the central target area. The maximum and minimum elevations in the target area are indicated by broken lines. Temperature changes measured in the target and rectal areas of the rats are plotted for model verification (*n* = 4). The gray solid line shows the average of each measured value and the gray bars show 95% confidence interval. The computed blood temperature is compared with the measured rectal temperature.

Figure 7 shows the temperature elevations in the target area rectum for each BASAR. Figure 7a–e shows the temperature in the target area for each BASAR. The black bold line shows the temperature elevation computed in the central target area. The target area consists of 249 cells, from which the minimum and maximum temperature elevations were extracted and indicated by the broken lines. To confirm the validity, the temperature computed using the numerical rat model was compared with the measured temperature values of the living rats (*n* = 4 for each BASAR). Following a few minutes of initial exposure, the brain temperature elevated rapidly and then linearly during exposure at 6 GHz (Figure 7b,c), and is almost constant at 10 GHz (Figure 7d,e). The linear increment is attributed to increased blood temperature. The temperature elevation did not reach a steady state at 1.5 GHz because the exposure time was short (Figure 7a). After the MW exposure, the target area temperature started to decrease rapidly and remained approximately 1.0 °C higher than the pre-exposure. The time course of the computed temperature elevation in the target agreed well with the measured temperature during and following MW exposure.

As shown in Figure 7f–j, the measured rectal temperature elevated linearly during exposure and remained almost constant after the exposure ended. The bar representing 95% confidence interval of measured temperature was wide following the MW exposure.

## 4. Discussion

In this study, parameters related to thermophysiological responses for MW exposures were estimated using an electromagnetic-thermodynamics simulation technique. The results indicated that the improved modeling parameters led to a computed temperature elevation that matched well with experimental measurements designed for the local exposure of the rat brain.

As described in the previous report [17], specific experimental conditions were set up in the current study to focus on local physiological changes caused by the localized MW exposure of the cortex. First, we improved the localization of MW exposure of the cortex by using two types of antenna. The exposure intensity in the cortex target area was estimated to be 16 or more times greater than that in the animal’s entire body. This facilitated in maintaining the exposure of the entire rat body at a low level. In particular, even the maximum-level MW exposure at 106 W·kg^−1^ of BASAR caused a very small temperature increase (<0.9 °C) in the rectum. Furthermore, in the exposure at 10 GHz, no significant temperature elevation was found in the rectum, whereas target area temperature elevated after the exposure. Thus, it is likely that the physiological responses in this study were obtained in the near absence of direct heating of the body, including heat generation at the body surface.

Second, temperature stabilization in each measured region was achieved under sham-exposed conditions. In physiological experiments using rodents, rectal temperature is maintained at 37 °C by using a feedback-regulated heating pad. However, this temperature regulation was unsuitable for our study because MW-related temperature changes would be compensated by it. The present experimental conditions were determined after evaluation of several factors such as heated pad shape, steady temperature, anesthesia type, and experimental duration. As shown in Figure 2 and Table 4, no significant mean temperature elevation from the initial rectal temperature was observed over the period of sham exposure; however, this did not hinder the detection of the small changes in temperature between exposed and sham-exposed groups during MW exposure.

Third, the rat’s target area temperature was kept below 39 °C throughout the experiment for all exposure conditions. This was attributed to the initial temperature of approximately 30 °C in the target area. Thus, severe toxic effects such as cell death and higher edema, observed in brain heated at around 42 °C [42], were unlikely to occur under the present conditions.

In contrast, several researchers found that cerebral hypothermia modified physiological functions such as neuronal activity [43] and blood–brain barrier [44]. However, it is known to be difficult to keep the rat cortex temperature at 37 °C in an anesthetized small animal. In particular, in our experiment, removal of parietal skin and exposure of a part of the skull were needed to provide localized MW exposure, leading to the lower initial temperature. Therefore, there might be limitations to some physiological functions. Nevertheless, it was interesting that the thermoregulation in the cortex was activated and its behavior was mimicked in the computational model we improved, even under the present limited conditions.

The parameters related to brain and rectal temperatures (*F_BB_* and *F_RB_*) were determined from the measured values of 6 and 10 GHz. The parameters used in our previous study [9] were arbitrary and derived from a one-exposure condition. The parameters derived in the present study were demonstrated to be applicable to different exposure frequencies and systems. Specifically, *F_BB_* affected the initial rate of increase in brain temperature, and *F_RB_* influenced the linear gradient of brain temperature elevation by using blood temperature. The rate of increase in brain temperature was rapid with decreases in the *F_BB_* results. The coefficients related to blood flow changes in a biological system were not affected by the frequency of the external electromagnetic field but by the internal heat load (SAR). Therefore, parameters derived through the measurements at multiple frequencies can provide accuracy and generality with respect to parameter extraction.

Blood flow regulation was modulated through tissue temperature and aided in lowering the tissue temperature against heating load. As shown in Figure 4, the effectiveness of temperature reduction in the target area between the cases with and without thermoregulatory modeling was more remarkable at the higher BASAR conditions. This is because of the increase in the blood flow produced by the temperature elevation due to exposure (e.g., Equation (7) for brain tissue). Simultaneously, temperature elevation was also regulated by the blood flow elevation (Equation (2)); this is not considered in the model without thermoregulation. The computed accuracy of the temperature elevation improved when thermoregulatory modeling was considered (Table 3). Moreover, thermoregulation significantly influenced the brain temperature elevation, with increases in the BASAR. These results show that the modeling of thermophysiological response is essential for estimating regional brain temperature in rats. A potential reason for the larger difference in *I_ΔT_W_* at higher frequencies can be attributed to the location control of the antenna. Fine location control is required at higher frequencies for high focality.

At 1.5 GHz, the SAR distribution was higher in the brain and throat (Figure 5a). This is because the size of the rat was smaller than the size of the antenna, and the MW diffracted around the side of the rat’s head [10]. Conversely, SAR absorption was gradually localized at 6 and 10 GHz (Figure 5b,c) because the penetration depth of MW decreased as the frequency increased. The distributions of temperature elevation (Figure 6) were similar to those of SAR. The temperature elevation distribution was much smoother because of thermal diffusion and blood perfusion.

The models in the present study allowed for the thermoregulatory response of rCBF (Equation (7)) and provided estimates of brain temperature elevations that were in good agreement with the measured data (Figure 7a–e). As is widely known, the estimations of temperature and blood flow are highly dependent on the computational location in local exposure. Variations were observed in the computed values inside the target area (249 elements). However, the model value corresponded reasonably well with the experimental data. Therefore, the findings suggest that the thermoregulatory responses in the brain and rectal regions were efficiently modeled for localized MW exposure in this frequency band.

We investigated that the computed blood temperature elevation is a tool to help predict rat core (rectal) temperature elevation. This is because the computed blood temperature was changed to satisfy the thermodynamic law; the heat in the tissue is transferred to and stored in the blood. This works well in this study because of higher blood perfusion rate and smaller dimension of a rat compared to those than in human [9,36]. Typically, the computed rectal temperature elevations were in extremely good agreement with the measured rectal values.

One of the limitations of the computation is attributable to phantom development, in which an automatic classification algorithm is applied. The heat transfer between the lung and internal air is simplified; one reason for this is that the lung cannot be modeled from CT images. A single value of thermal conductivity, specific heat, and blood flow is assigned to a corresponding tissue throughout the body at the thermoneutral condition [45]. Nonetheless, the computation agreed well with measurement because we focused on the brain temperature elevation and blood flow. The heat diffusion length in the brain is of a few millimeters because of higher blood perfusion rate. In addition, the volume-averaged blood temperature is changed so as to satisfy the first law of the thermodynamics. We demonstrated the effectiveness of our multiphysics modeling, which is still simple to implement, by comparing with measure data. The main purpose of this study was to develop the model for rat brain and thus the result obtained here is not directly applied to humans. Thermophysiological modeling in humans can be found in references [46,47,48], although they are useful and validated for relatively small temperature elevation.

It remains unclear whether other physiological parameters such as heart rate and blood pressure could involve the regional temperature elevations including core body’s temperature. However, the topics are challenging and considered as the next step, because of several difficulties. As a first step, we focused on the relationship between target cortex temperature and rCBF for electromagnetic safety for localized exposure. That is the main reason why we developed the special antennas to eliminate the direct body heating under MW exposure. As the present results showed, even in our simpler model, the computed estimation matched well with the measured quantities during and after exposure in rats. This result may suggest that rCBF change is the dominant parameter to control the local temperature, by comparing with other physiological parameters. In the next step, we may have to discuss additional mechanism carefully.

## 5. Conclusions

In this study, parameters related to thermophysiological responses for MW exposures were estimated using an electromagnetic-thermodynamics simulation technique. Specifically, the parameters related to cerebral blood flow were estimated with a high degree of accuracy. The improved modeling parameters resulted in computed results that corresponded well with the measured values at a super-high-frequency band that was not previously computed. The computational model provides the basis to simulate MW exposure for thermal evaluation in a consistent model ranging from conventional frequencies to frequencies exceeding 6 GHz. This simulation can be helpful in estimating temperature elevation and changes in blood flow rate at multiple observation points that are difficult to measure, and can help analyze the physiological effects caused by microwave exposure and improve health risk assessment.

## Figures and Tables

**Table 1:** Dielectric properties of the rat model.

Tissues	1.5 GHz		6 GHz		10 GHz	
	*ε_r_*	*σ*	*ε_r_*	*σ*	*ε_r_*	*σ*
		(S·m^−1^)		(S·m^−1^)		(S·m^−1^)
Fat	11.1	0.16	9.8	0.87	8.8	1.71
Muscle	54.9	1.38	48.2	5.20	42.8	10.63
Bone	23.8	0.65	15.2	2.23	12.7	3.86
Eye	68.7	1.89	47.5	5.72	41.5	11.31
Brain	46.7	1.15	43.7	5.22	38.1	10.31
Skin	39.4	1.07	34.9	3.89	31.3	8.01

Relative permittivity is denoted as *ε_r_*, conductivity is denoted as *σ*.

**Table 2:** Thermal properties of the rat model.

Tissues	*ρ*	*K*	*C*	*B*_0_	*A*
	(kg·m^−3^)	(W·m^−1^·°C^−1^)	(J·kg·°C^−1^)	(W·m^–3^·°C^−1^)	(W·m^−3^)
Fat	916	0.25	3000	6299	4983
Muscle	1047	0.50	3800	2880	2278
Bone	1990	0.37	3100	12,317	9743
Eye	1009	0.57	4000	0	0
Brain	1038	0.57	3800	35,692	28,233
Skin	1125	0.42	3600	11,605	9180

Density is denoted as *ρ*, thermal conductivity is denoted as *K*, specific heat is denoted as *C*, basal blood flow parameter is denoted as *B*_0_, and basal metabolism is denoted as *A*.

**Table 3:** Comparison between experimental and computed data based on Equation (9).

f	BASAR	I_*ΔT_W/O_*	I_*ΔT_W_*	Reduction
(GHz)	(W·kg^−1^)	(%)	(%)	(percentage Point)
1.5	75	6.6	2.7	3.9
6	64	9.2	3.7	5.5
	106	19.3	4.7	14.6
10	29	10.7	7.2	3.5
	79	18.7	5.3	13.4

Temperature elevations with thermoregulation Δ*T_w_* and without thermoregulation Δ*T_w/o_* are shown.

**Table 4:** Temperature conditions in the experiment.

f (GHz)	BASAR (W·kg^−1^)	Target Temperature (°C)		Rectal Temperature (°C)	
		Before	After	Before	After
1.5	0	33.3 ± 0.1	33.5 ± 0.1	37.1 ± 0.1	37.2 ± 0.1
	75	33.0 ± 0.2	36.2 ± 0.2 *	37.4 ± 0.1	37.9 ± 0.0 *
6	0	30.3 ± 0.3	31.1 ± 0.2	37.5 ± 0.2	37.6 ± 0.3
	64	30.9 ± 0.6	35.6 ± 0.6 *	37.0 ± 0.2	37.6 ± 0.2
	106	30.6 ± 0.1	37.6 ± 0.2 *	37.3 ± 0.2	38.2 ± 0.1 *
10	0	30.2 ± 0.6	30.4 ± 0.2	36.7 ± 0.2	36.7 ± 0.2
	29	30.1 ± 0.3	33.6 ± 0.4 *	36.4 ± 0.2	36.5 ± 0.1
	79	29.4 ± 0.3	36.5 ± 0.2 *	36.5 ± 0.1	36.7 ± 0.0

Regional temperatures in target area and rectum before and after the MW exposure were compared. The temperature shown as the mean ± SEM. *: *p* < 0.05 vs. before the MW exposure.
